# 1820. Source Control and Odontogenic Infections in an Urban Safety Net Hospital: What, When and How?

**DOI:** 10.1093/ofid/ofad500.1649

**Published:** 2023-11-27

**Authors:** Erin Pollock, Mohamed Elbathani, Anirudh Damughatla, Lea M Monday

**Affiliations:** Wayne State University School of Medicine, Detroit, Michigan; Wayne State University School of Medicine, Detroit, Michigan; Wayne State University School of Medicine, Detroit, Michigan; Wayne state University School of Medicine, Detroit, Michigan

## Abstract

**Background:**

Odontogenic infections cause 2.2 million emergency department (ED) visits annually. Infectious diseases (ID) physicians are frequently consulted and recommend source control procedures by oral and maxillofacial surgery (OMFS). However, patients are often instructed to follow-up outpatient which may be difficult due to social determinants of health. Despite this common occurrence, almost no studies exist in the ID literature on follow-up rates or outcomes of these management strategies. We aim to identify and characterize patients with odontogenic infections, and determine whether definitive source control (SC) impacts outcomes.

**Methods:**

An observational cohort study analyzing patients seen by OMFS for odontogenic infection over 14 months (1/2022-2/2023) at a safety net hospital system in Detroit, MI. Odontogenic infection was defined as that of the jaw, neck, or face originating from a tooth or its supporting structures. Non-infectious consults were excluded (Fig 1). Characteristics and outcomes were compared between patients with and without SC. Primary outcome was incidence of 30-day readmission. Mortality and microbiologic data were also collected.

**Figure:**
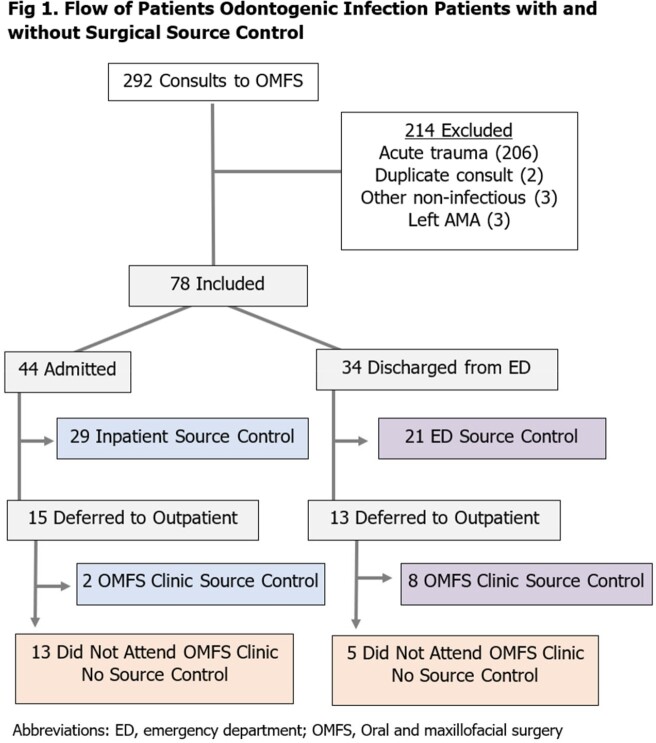

**Results:**

78 patients were evaluated by OMFS; 44 (56%) were admitted and 34 (44%) were discharged from the ED. SC occurred in 60 patients, 50 of which had SC on the index admission or ED visit (29 of 44 admitted and 21 of 34 ED patients, respectively) (Fig 1). SC was deferred in 28 patients, 10 of whom did later receive it. Follow up rates for ED patients who SC deferred were high (8 of 13 patients). Only 2 of 15 deferred inpatients were seen (Fig 1). No documented SC occurred in 18 patients. Groups did not differ significantly in clinical characteristics, antibiotics, or rates of ID consultation (Table 1). There was a trend for SC patients to be male (p=0.057) and require intensive care unit support (p=0.081) (Table 1). Readmission rates were higher in those without source control (28% versus 3%, p=0.001). Common pathogens isolated included oral *Streptococci* and gram-positive rods (Fig 2).

**Figure:**
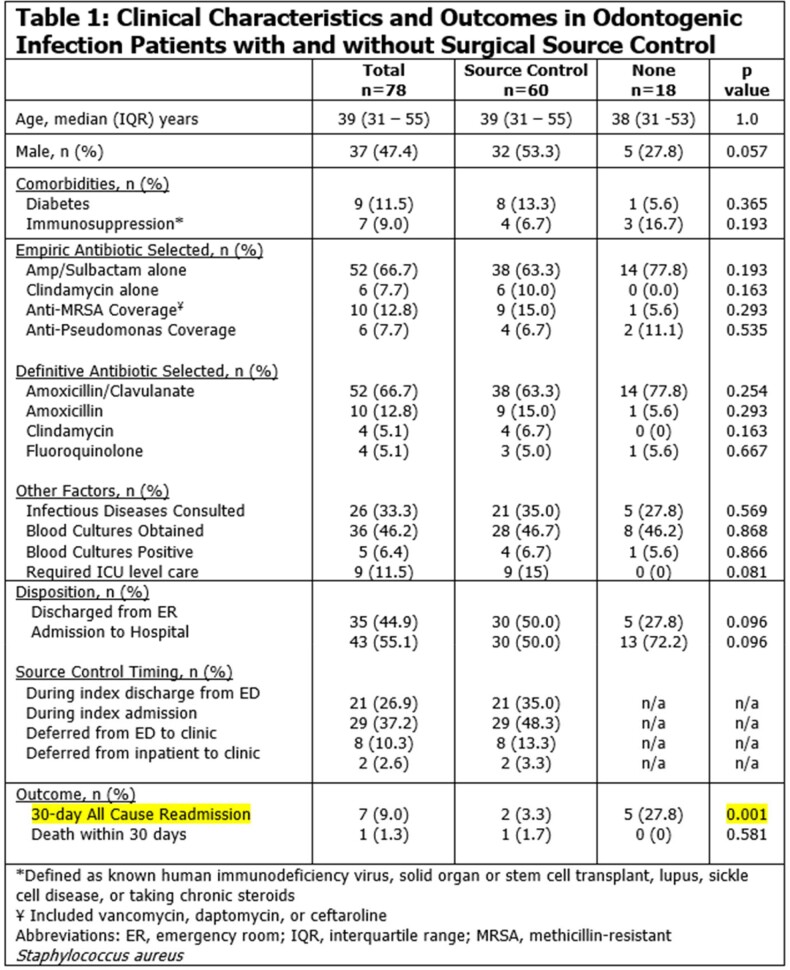

**Figure:**
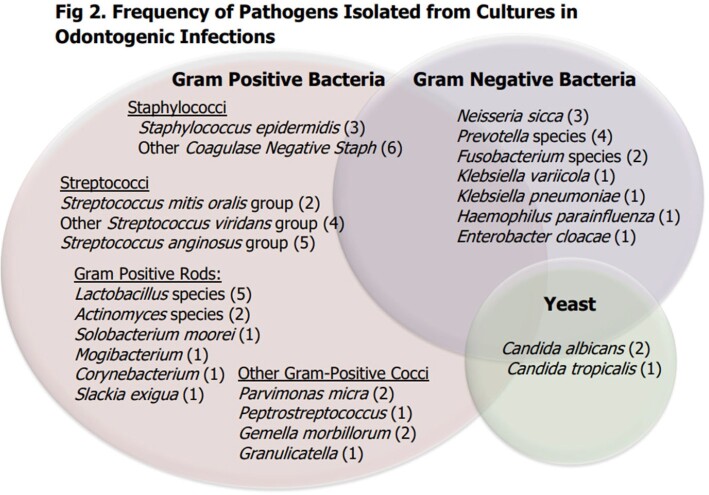

**Conclusion:**

Odontogenic infection patients without source control had high rates of readmission despite being less critically ill. The ID community should take a more active role in studying the optimal management strategy for this common occurrence.

**Disclosures:**

**All Authors**: No reported disclosures

